# Multi-Omics and Experimental Validation Reveal Anti-HCC Mechanisms of Tibetan Liuwei Muxiang Pill and Quercetin

**DOI:** 10.3390/ph18060900

**Published:** 2025-06-16

**Authors:** Wei Chen, Yanzhen Li, Chuting Zhang, Hang Zhou, Jun Ma, Deep K. Vaishnani, Bingchi Zeng, Jinglu Yu, Huchao Mao, Jianjian Zheng

**Affiliations:** 1 Zhejiang Key Laboratory of Intelligent Cancer Biomarker Discovery and Translation, The First Affiliated Hospital of Wenzhou Medical University, School of Laboratory Medicine and Life Sciences, Wenzhou Medical University, Wenzhou 325000, China; 15657057690@163.com (W.C.); 18858737988@163.com (Y.L.); drzh0717@163.com (H.Z.); yujinglu2482@163.com (J.Y.); m1luku147@163.com (H.M.); 2 Renji College, Wenzhou Medical University, Wenzhou 325035, China; 17858052691@163.com; 3 School of Stomatology, Wenzhou Medical University, Wenzhou 325035, China; zcting20@163.com; 4 Department of Pathology, Wenzhou Medical University, Wenzhou 325035, China; martinskyer@163.com; 5 School of International Studies, Wenzhou Medical University, Wenzhou 325035, China; 17815790832@163.com

**Keywords:** hepatocellular carcinoma, Liuwei Muxiang pills, bioinformatics, traditional Tibetan medicine, high-resolution mass spectrometry

## Abstract

**Background**: Hepatocellular carcinoma (HCC) remains a significant therapeutic challenge due to its complex molecular mechanisms and limited effective treatments. Although the Tibetan medicine Liuwei Muxiang Pill (LWMX) has shown efficacy in gastric and colorectal cancer, no study has yet demonstrated its potential anti-HCC activity. Objective: To evaluate the therapeutic effects of LWMX on HCC. **Methods**: Integrating network pharmacology, high-resolution mass spectrometry, machine learning, and molecular dynamics simulations, combined with in vitro experiments for mechanism validation. Results: The study identified (Checkpoint kinase 1) CHK1 as a key target of LWMX in HCC regulation and confirmed that quercetin can form a stable complex with CHK1. In vitro experiments demonstrated that LWMX and its active component quercetin significantly inhibit the proliferation and migration of HCC cells. **Conclusions**: The study establishes CHK1 as a key therapeutic target, confirming the potential of LWMX and its core component quercetin in the treatment of HCC.

## 1. Introduction

Hepatocellular carcinoma (HCC) is among the most common malignant tumors worldwide, exhibiting consistently high incidence and mortality rates, especially in Asia and Africa [1]. Recent advancements in the diagnosis and treatment of HCC have not significantly improved therapeutic outcomes, which continue to be suboptimal due to the complex molecular mechanisms and high heterogeneity of the disease. A deeper exploration of the molecular mechanisms underlying HCC and the identification of effective therapeutic strategies have emerged as central themes in contemporary research.

Traditional Chinese medicine (TCM) exhibits distinct benefits in cancer treatment, especially regarding its capacity for multi-target and multi-pathway regulation [2]. The Tibetan herbal formula “Liuwei Muxiang Pill” (LWMX) is a traditional compound medicine utilized extensively for the treatment of various diseases [3,4]. However, its mechanism of action in HCC therapy has not been systematically investigated. Notably, one of its active components, quercetin, a natural flavonoid, has been shown to possess diverse biological activities, including anti-inflammatory, antioxidant, and anti-tumor effects. Nevertheless, the specific mechanisms of quercetin in HCC, including its impact on autophagy and macrophage polarization and its synergistic effects with LWMX, warrant further exploration [5,6].

Recent advancements in network pharmacology, high-resolution mass spectrometry, machine learning algorithms, molecular docking, and dynamic simulation technologies have equipped researchers with effective tools for elucidating the multi-target mechanisms of traditional Chinese medicine formulations [7,8,9,10]. Integrating multi-omics data enhances the understanding of the intricate interactions between drugs and diseases, providing valuable insights for drug development and personalized therapy.

This research investigates the mechanisms by which LWMX and its active component quercetin may contribute to the treatment of HCC, employing a multi-dimensional systems biology approach. Initially, we utilized network pharmacology and machine learning algorithms to identify shared targets of LWMX and HCC, subsequently constructing a protein–protein interaction network. Molecular docking and dynamic simulations were employed to validate the binding modes of quercetin with key targets. A machine learning-based prognostic prediction model for HCC was developed utilizing multi-gene expression data. The inhibitory effects of LWMX and quercetin on HCC cell proliferation and migration were experimentally validated.

This series of investigations elucidates the potential molecular mechanisms of LWMX and its active components in HCC therapy, while also providing new theoretical and experimental evidence for the development of TCM-based therapeutic strategies for HCC. This study’s findings are anticipated to provide new insights for personalized treatment and drug development in HCC, while also making a substantial contribution to the advancement of TCM research.

## 2. Results

### 2.1. The Acquisition of Intersection Genes Between LWMX and HCC

**Figure 1:**
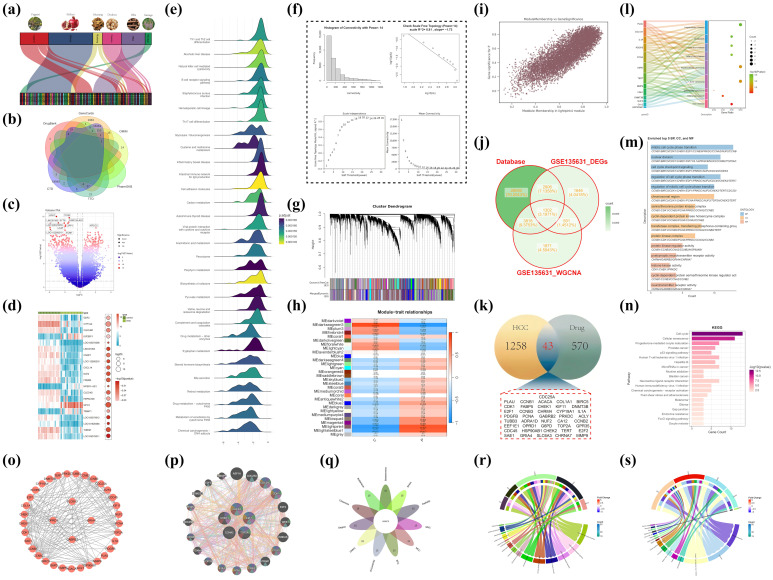
Molecular mechanisms of Liuwei Muxiang Pill in hepatocellular carcinoma treatment. (**a**) Sankey diagram depicting the relationship between LWMX components and their compounds. (**b**) Integration of databases for HCC-related genes. (**c**,**d**) Differentially expressed genes in HCC. (**e**) GSEA of upregulated and downregulated genes. (**f**) Co-expression network optimization. (**g**,**h**) Hierarchical clustering and HCC-related gene modules. (**i**) GS-MM analysis of gene significance and module membership. (**j**) Overlapping genes in HCC pathogenesis. (**k**) Overlapping genes between LWMX and HCC. (**l**) DO analysis of overlapping genes. (**m**) GO enrichment analysis of LWMX in HCC treatment. (**n**) KEGG pathway analysis of overlapping genes. (**o**) Protein–protein interaction network. (**p**) Functions of key hub genes. (**q**) Identification of key hub genes. (**r**,**s**) GO and KEGG analyses of hub genes.

The top eight compounds were successfully identified, including dehydrocostus lactone, ellagic acid, quercetin, luteolin, kaempferol, and piperine. To validate these components further, the structures of the compounds were obtained from the PubChem database and are presented in Figure S2 of the Supplementary Material. Through the integration of database and bioinformatics methodologies, the intersection genes of LWMX and HCC were identified, demonstrating a significant association with the cell cycle in the pathogenesis and progression of HCC. A Sankey diagram (Figure 1a) illustrates the relationship among the six main drug components in LWMX (Yuganzi, Shiliuzi, Muxiang, Doukou, Biba, Baxiaga) and their respective compounds. The integration of six databases resulted in the identification of 36,617 genes associated with HCC (Figure 1b). Analysis of the GSE135631 dataset revealed 6444 differentially expressed genes (DEGs) (Figure 1c,d). Gene Set Enrichment Analysis (GSEA) indicated that upregulated genes were primarily enriched in pathways related to the cell cycle and DNA repair, whereas downregulated genes were significantly linked to drug metabolism enzymes and bile acid synthesis (Figure 1e). The co-expression network demonstrated optimal performance at a power value of 14 (Figure 1f). Several functional gene modules were identified, with the pink module exhibiting the highest correlation with HCC phenotypes (r = 0.82, Figure 1g,h). The Gene Significance-Module Membership (GS-MM) analysis corroborated the positive correlation between gene significance and module membership, as illustrated in Figure 1i. Intersection analysis identified 1302 conserved genes across datasets, which may serve as core regulators of HCC pathogenesis (Figure 1j).

A comparative analysis of the drug components of LWMX and HCC-related target genes identified 43 overlapping genes (Figure 1k). Analysis of the Disease Ontology (DO) revealed that these genes are predominantly linked to liver diseases (Figure 1l). GO enrichment analysis revealed significant enrichment in biological processes (BP) related to “mitotic cell cycle transition” and “cell cycle checkpoint signaling.” Cellular components (CC) were primarily associated with “chromatin regions” and “serine/threonine protein kinase complexes.” Molecular functions (MF) were critical in “protein kinase regulation” and “histone kinase activity” (Figure 1m). KEGG pathway analysis confirmed the significant enrichment of these genes in the “cell cycle,” “cell senescence,” and “p53 signaling pathway” (Figure 1n). Analysis of the protein–protein interaction network revealed five hub genes (E2F1, CCNA2, CHK1, TOP2A, CHK2) via topological parameter screening (Figure 1o,q). The genes are associated with cell cycle checkpoints, the G1/S phase transition, and the negative regulation of the mitotic cell cycle (Figure 1p). Subsequent GO and KEGG analyses validated the pivotal function of these hub genes in regulating the cell cycle (Figure 1r,s).

### 2.2. Unveiling the Nuclear Role and Interactions of Hub Genes in HCC

**Figure 2:**
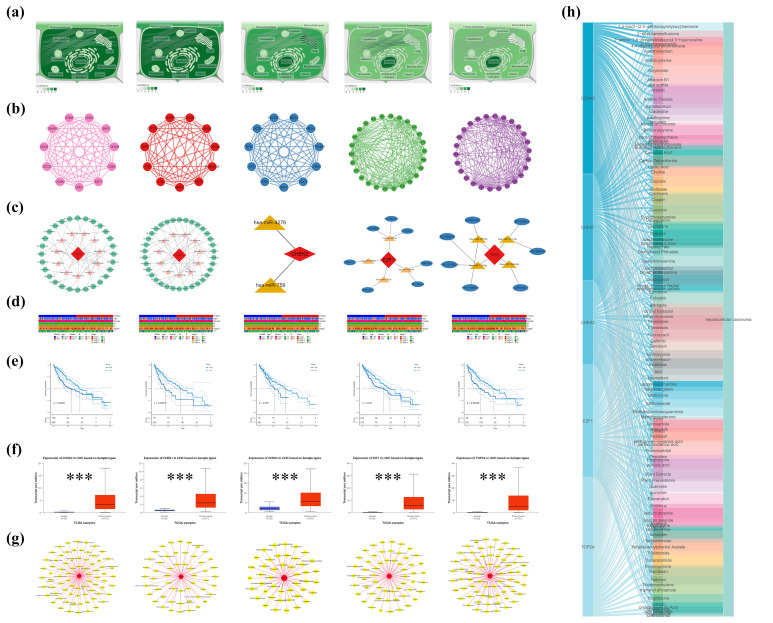
Localization and interaction networks of key genes in hepatocellular carcinoma. (**a**) Localization of CCNA2, CHK1, CHK2, E2F1, and TOP2A in the cell nucleus. (**b**) Protein–protein interaction network showing direct and indirect connections. (**c**) ceRNA regulatory network. (**d**) Correlation of gene expression with clinical parameters. (**e**) Survival analysis of gene expression. (**f**) Expression-level comparison between cancer and normal tissues. (**g**) “Protein-hepatocellular carcinoma-environmental chemical” network. (**h**) Sankey diagram of environmental chemical and gene interactions. * *p* < 0.05, ** *p* < 0.01, *** *p* < 0.001.

Analysis of subcellular localization indicated that five essential genes—CCNA2, CHK1, CHK2, E2F1, and TOP2A—are located in the cell nucleus (Figure 2a). Five protein–protein interaction networks were constructed, demonstrating both direct and indirect connections among each gene and other genes (Figure 2b). Post-transcriptional regulation networks were delineated through mRNA-miRNA interaction analysis, which identified 39, 41, 2, 21, and 22 regulatory miRNAs targeting CCNA2, CHK1, CHK2, E2F1, and TOP2A, respectively. Subsequent interrogation of the sponge-scanning database identified 28, 35, 0, 7, and 7 miRNA-lncRNA pairs. The integration of multi-omics data resulted in the construction of a ceRNA network consisting of 112 nodes and 189 edges, illustrating coordinated regulation via 17 shared miRNAs and 9 hub lncRNAs (Figure 2c).

This study examines the clinical relevance of these genes in hepatocellular carcinoma by analyzing their correlation with clinical parameters, including tumor stage and size (Figure 2d). The findings demonstrate that CCNA2, CHK1, E2F1, and TOP2A exhibit significant associations with tumor stage and size, whereas CHK2 shows a correlation solely with age. Survival analysis indicated that the expression levels of CCNA2, CHK1, E2F1, and TOP2A are significantly associated with the survival prognosis of hepatocellular carcinoma patients, with *p*-values of 0.0038, 0.00013, 0.013, and 0.00066, respectively. In contrast, the expression of CHK2 did not demonstrate a significant correlation with survival prognosis (*p* = 0.067) (Figure 2e). A comparison of gene expression levels between hepatocellular carcinoma tissues and normal tissues demonstrated significant differences (*p*-value < 0.0001) (Figure 2f). Five genes exhibited significant upregulation in hepatocellular carcinoma cell lines (*p* < 0.05, Supplementary Figure S4).

A network illustrating the interaction among these genes and environmental chemicals, termed “protein-hepatocellular carcinoma-environmental chemical,” was constructed (Figure 2g) to enhance understanding. This network elucidates established risk factors for HCC while also identifying novel potential environmental chemicals, including acrylamide, diethylnitrosamine, and estradiol, which are acknowledged as endocrine disruptors in the environment. A Sankey diagram (Figure 2h) illustrates the intricate interactions among these chemicals and target genes.

### 2.3. Molecular Docking Analysis of the Core Active Ingredients of LWMX with Hub Genes

The molecular docking study aimed to evaluate the binding potential of four active components from Tibetan medicine with five core target proteins. The literature (doi: 10.3390/ph17040429) reports the core active ingredients of the Tibetan medicine Liuwei Muxiang Pill, which are quercetin, (-)-epigallocatechin-3-gallate, luteolin, and palmitoleic acid [3,11]. Epigallocatechin gallate (EGCG), luteolin, palmitoleic acid, and quercetin were docked with CCNA2, CHK1, CHK2, E2F1, and TOP2A, respectively. The predicted binding energies (kcal/mol) are provided in Supplementary Material Table S3.

**Figure 3:**
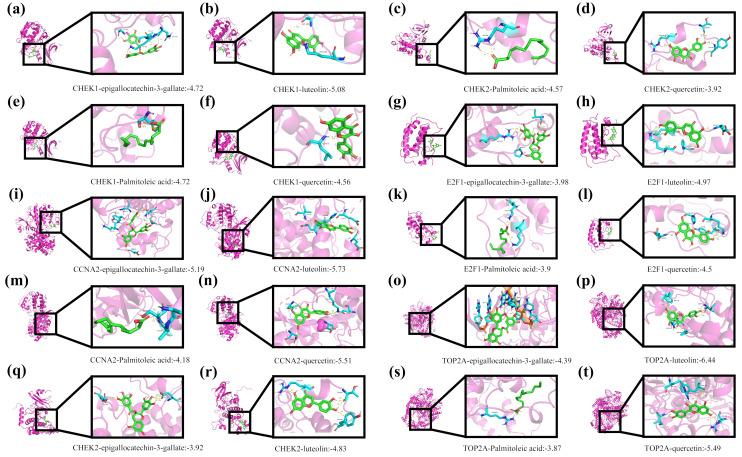
Molecular docking of LWMX components with hub genes. (**a**–**t**) Binding poses and energies of quercetin, EGCG, luteolin, and palmitoleic acid to CHK1, CCNA2, CHK2, TOP2A and E2F1.

As illustrated in Figure 3a–t, all four active components exhibited some degree of binding affinity (negative binding energy values) with the five core target proteins. Among them, quercetin showed a relatively stronger binding tendency with CHK1 (ΔG = −4.56 kcal/mol), as indicated by its lower binding energy. Additionally, quercetin also demonstrated favorable binding potential with CHK2 (ΔG = −3.92 kcal/mol). Both EGCG and luteolin displayed moderate binding energies across multiple targets. In contrast, palmitoleic acid exhibited relatively higher binding energies (smaller absolute values) across all targets, suggesting weaker binding affinity with these proteins.

These preliminary docking results imply that the four active components of Tibetan medicine may exert their biological effects by targeting the five core proteins—CCNA2, CHK1, CHK2, E2F1, and TOP2A.

### 2.4. CHK1 Is a Core Gene with Diagnostic and Prognostic Value

**Figure 4:**
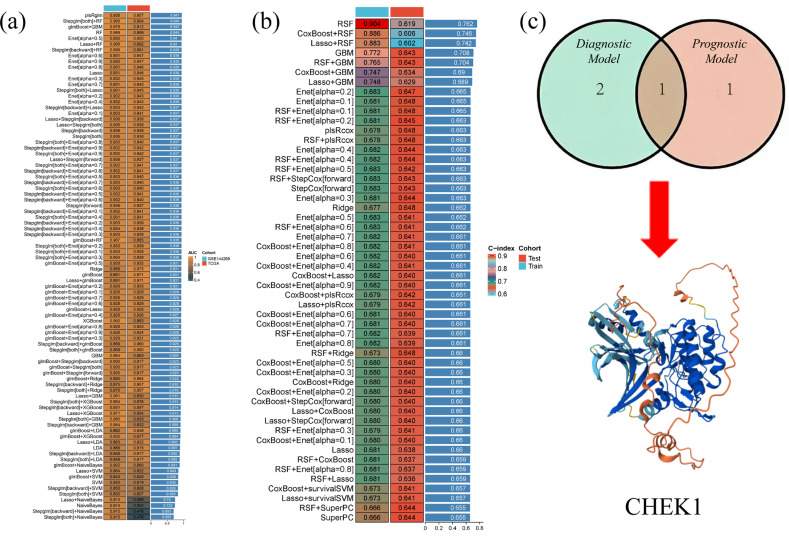
Algorithm performance comparison and key gene identification. (**a**) plsRglm algorithm exhibits optimal performance. (**b**) RSF algorithm demonstrates best prognostic predictive efficacy. (**c**) CHK1 was identified as a core gene with diagnostic and prognostic value.

During the construction of the diagnostic model, the plsRglm algorithm performed the best, achieving an AUC value of 0.947 (Figure 4a). In the construction of the prognostic model, the RSF algorithm performed the best, with a C-index of 0.762 (Figure 4b). Through this approach, the CHK1 gene was successfully identified (Figure 4c). The CHK1 gene demonstrated importance in both models, ranking second in the plsRglm model and first in the RSF model, indicating its diagnostic and prognostic value. In the external datasets GSE39791 and GSE76427, the AUC values of the ROC curve for the CHK1 gene were 0.837 and 0.883, respectively (Supplementary Material Figure S3).

### 2.5. Role and Correlation of CHK1 in the Immune Microenvironment

**Figure 5:**
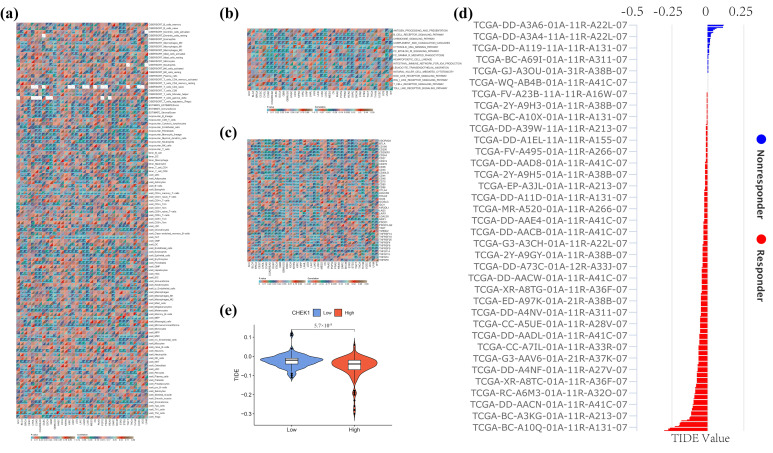
CHK1 correlation in HCC immune microenvironment. (**a**) CHK1’s significant associations with immune cells, particularly T cells in HCC. (**b**) Immunoinflammatory analysis. (**c**) Correlations with immune checkpoint genes, including positive with ADORA2A, CTLA4, HLA2, and negative with PD1LG2, TIMG2. (**d**,**e**) TIDE score differences suggest CHK1’s role in tumor immune evasion. * *p* < 0.05, ** *p* < 0.01, *** *p* < 0.001.

The relationship between CHK1 and diverse immune cells within the pan-cancer immune microenvironment demonstrates notable associations with several immune cell types (Figure 5a). This study examines CHK1’s role in the immune microenvironment of HCC, with the HPA database indicating predominant CHK1 expression in T cells within HCC, leading to a specific focus on T-cell infiltration. CIBERSORT analysis demonstrated a positive correlation between CHK1 expression in hepatocellular carcinoma (HCC) and CD4+ memory-activated T cells (r = 0.122, *p* = 0.018), as well as follicular helper T cells (r = 0.233, *p* < 6.16 × 10^−6^). In contrast, estimation algorithms exhibited negative correlations with estimated scores (r = −0.202, *p* < 9.10 × 10^−5^) and stromal scores (r = −0.329, *p* < 9.53 × 10^−11^), indicating possible immunosuppressive effects. xCell analysis confirmed the correlation of CD4+ memory T cells (r = 0.254, *p* < 7.76 × 10^−7^).

Pathway analysis reveals a significant negative correlation with the B-cell receptor signaling pathway (r = −0.306, *p* = 1.66 × 10^−10^), followed by the chemokine signaling pathway (r = −0.273, *p* = 1.40 × 10^−8^) (Figure 5b). Notable immune checkpoint correlations consist of positive associations with ADORA2A, CTLA4, and HLA2, alongside negative associations with CD160, CD200R1, and CD244 (Figure 5c). CHK1 exhibits a negative correlation with PD1LG2/TIMG2 (*p* < 1 × 10^−7^) and a positive correlation with TNFRSF4 (*p* < 1 × 10^−13^) and TNFRSF9 (*p* < 1 × 10^−5^). A comparative analysis of TIDE scores indicates significant intergroup differences (*p* ≈ 5.7 × 10^−8^), implying that CHK1 may influence tumor immune evasion via increased TIDE scores (Figure 5d,e). The Supplementary Material Table S3 showed that CHK1-related genes were significantly enriched in the JAK-STAT pathway, and STAT3 was a key component of this pathway. This was consistent with the previously reported mechanism that CHK1 regulates PD-L1 through STAT3, suggesting that CHK1 might regulate PD-L1 expression via the JAK-STAT/STAT3 pathway and participate in tumor immune evasion.

### 2.6. Analysis Results of the Association Between CHK1 and Drug Sensitivity in HCC

**Figure 6:**
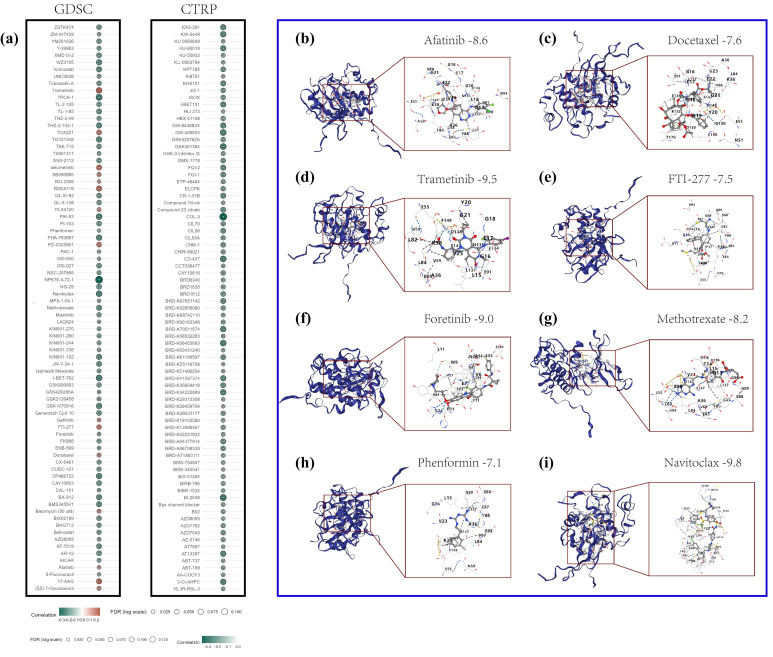
CHK1 correlation with drug sensitivity and molecular docking binding energies in HCC. (**a**) CHK1 correlation with GDSC and CTRP drug sensitivity, with red for positive and green for negative correlation. (**b**–**e**) Binding energies for positively correlated drugs: Afatinib (−8.6 kcal/mol), Docetaxel (−7.6 kcal/mol), Trametinib (−9.5 kcal/mol, highest), FTI-277 (−7.5 kcal/mol). (**f**–**i**) Binding energies for negatively correlated drugs: Foretinib (−9.0 kcal/mol), Methotrexate (−8.2 kcal/mol), Phenformin (−7.1 kcal/mol), Navitoclax (−9.8 kcal/mol, highest).

This study examines the relationship between CHK1 expression and drug sensitivity in hepatocellular carcinoma (HCC). It presents correlation analyses of CHK1 expression and drug response profiles sourced from the Genomics of Drug Sensitivity in Cancer (GDSC) and Cancer Therapeutics Response Portal (CTRP) databases, with red and green indicating positive and negative correlations, respectively (Figure 6a). The molecular docking binding energies of the selected drugs exhibiting positive correlations with the CHK1 protein are as follows: Afatinib −8.6 kcal/mol, Docetaxel −7.6 kcal/mol, Trametinib −9.5 kcal/mol, and FTI-277 −7.5 kcal/mol (Figure 6b–e). Trametinib demonstrates the highest binding energy, indicating the most robust interaction with the CHK1 protein. For drugs exhibiting negative correlations, the molecular docking binding energies for Foretinib, Methotrexate, Phenformin, and Navitoclax are −9.0, −8.2, −7.1, and −9.8 kcal/mol, respectively. Notably, Navitoclax possesses the highest binding energy, suggesting its potential to inhibit CHK1 activity (Figure 6f–i).

### 2.7. Analysis of Structural Stability and Dynamic Changes of CHK1 and Quercetin

**Figure 7:**
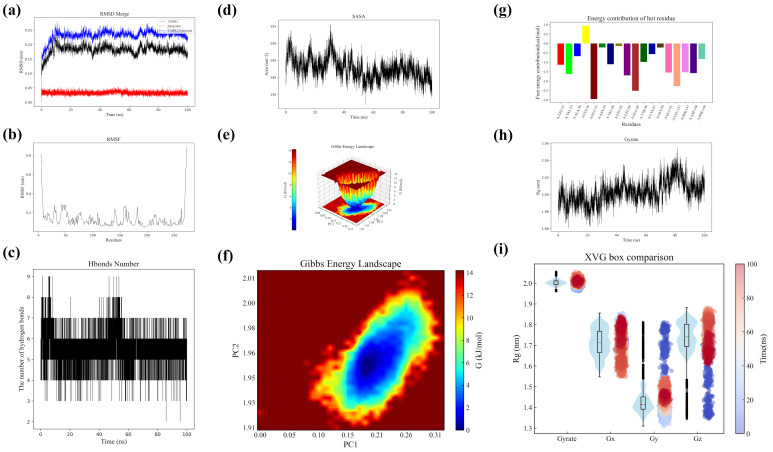
MD analysis of CHK1-quercetin interaction. (**a**) RMSD: CHK1 stabilizes at ~0.15 nm; quercetin remains rigid (~0.05 nm). Complex peaks at ~0.25 nm, indicating reorganization. (**b**) RMSF: Localized flexibility near active site; quercetin maintains global stability. (**c**) H-bonds: Stable interactions highlight strong binding affinity. (**d**) SASA: CHK1 fluctuates (135–155 nm^2^); quercetin reduces volatility. (**e**,**f**) Free energy: Two low-energy states; quercetin favors optimized conformation. (**g**) Residue energy: A:LYS38 stabilizes; A:GLU55/A:LEU85 hinder binding. (**h**,**i**) Rg: CHK1 transitions to expanded state, stabilizing with quercetin-induced rigidity.

We analyzed root mean square deviation (RMSD) trajectories to assess the structural dynamics of CHK1 and its interaction with quercetin. The CHK1 protein (black curve) demonstrates a swift initial increase in RMSD, stabilizing at approximately 0.15 nm, indicative of structural modifications preceding equilibrium. Quercetin (red curve) exhibits a stable RMSD of approximately 0.05 nm, signifying structural rigidity. The CHK1-quercetin complex exhibits an initial peak at approximately 0.25 nm, followed by stabilization at higher levels, indicating structural reorganization (Figure 7a). Analysis of protein RMSF indicates general conformational stability, accompanied by localized flexibility in particular residues adjacent to the active site. Quercetin binding results in minor fluctuations while maintaining overall structural integrity (Figure 7b).

Throughout the simulation, multiple stable hydrogen bonds between CHK1 and quercetin are maintained, primarily involving critical residues. Fluctuations are observed but persist at elevated levels, indicating a strong binding affinity (Figure 7c). CHK1’s solvent-accessible surface area (SASA) varies between 135 and 155 nm^2^ following quercetin binding, indicating transient fluctuations without a net structural expansion. Quercetin reduces SASA volatility in comparison to ligand-free conditions (Figure 7d). Free energy landscapes reveal two low-energy conformational states with negligible energy differences, indicating a dynamic equilibrium. Quercetin selectively associates with a conformation that is optimized for electrostatic and hydrophobic interactions (Figure 7e,f).

Residue-specific free energy contributions indicate A:LYS38 serves as a favorable binding site, whereas A:GLU55 and A:LEU85 impede stability. Most residues demonstrate neutral effects (Figure 7g). The radius of gyration (Rg) of CHK1 transitions from a compact state (0–20 ns) to an expanded state (20–80 ns), ultimately stabilizing at a higher equilibrium (80–100 ns). Temporal Rg distributions demonstrate a progressive structural compaction, especially in the Gx/Gy directions, influenced by quercetin-induced rigidity (Figure 7h,i). MD simulations indicate that quercetin improves the structural compactness and stability of CHK1 through specific interactions, providing insights into its regulatory mechanism and therapeutic potential.

### 2.8. Selecting Quercetin for Validation: Superior Affinity, Stability in HCC

This study selected quercetin as the primary active ingredient in Liuwei Muxiang Pill for experimental validation due to several key reasons: Molecular docking results demonstrate that quercetin shows considerable affinity for the CHK1 protein (ΔG = −4.56 kcal/mol), offering initial theoretical evidence for its potential bioactivity. Molecular dynamics simulations additionally validated the structural stability of the quercetin-CHK1 complex. In the 100 ns simulation, the RMSD of the complex stabilized at 0.25 nm, accompanied by the formation of a stable hydrogen bond network, with key residues such as A:LYS38 playing a significant role. The findings indicate that quercetin may facilitate conformational optimization of CHK1, which enhances the stability of the complex and potentially contributes to its antitumor effects. Quercetin, a natural flavonoid, has been extensively documented for its anticancer properties, including the induction of cell cycle arrest and the promotion of apoptosis. The findings are consistent with prior enrichment analysis results, which emphasized the strong association of key targets with the cell cycle. Consequently, the choice of quercetin for experimental validation facilitates a thorough examination of its antitumor mechanisms and investigates its potential applications in the treatment of HCC.

### 2.9. CHK1 Overexpression in HCC Detected

**Figure 8:**
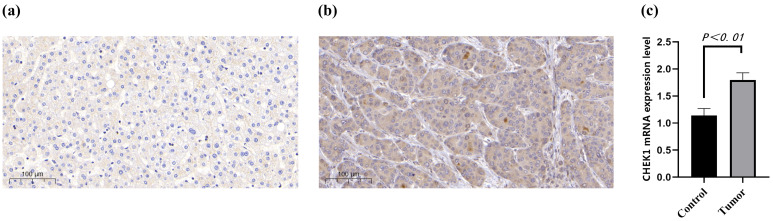
Immunohistochemical analysis of CHK1 expression in HCC and normal tissue. (**a**) Negative CHK1 expression in normal tissue. (**b**) Positive CHK1 expression in HCC tissue, with brownish-yellow granules in cytoplasm and nuclei. (**c**) Comparison showing significantly higher CHK1 expression in HCC samples versus normal liver tissue, indicating CHK1’s role in HCC development.

Through immunohistochemical analysis, the overexpression of CHK1 in HCC and its distribution in the surrounding normal tissue were identified. The brownish-yellow granules were distributed in the cytoplasm and nuclei. Overall, CHK1 expression in normal tissue was negative (Figure 8a). In HCC tissue, CHK1 expression was positive (Figure 8b). The expression of CHK1 in HCC samples was significantly higher than that in normal liver tissue, confirming that CHK1 plays a significant role in the occurrence and development of HCC (Figure 8c). The results demonstrated that the mRNA level of CHK1 in HCC was significantly decreased after treatment with Tibetan medicine Liuweimuxiang Pills compared to the control group (*p* < 0.001; Supplementary Material Figure S6).

### 2.10. LWMX & Quercetin Inhibit Huh7 Cell Proliferation and Migration

**Figure 9:**
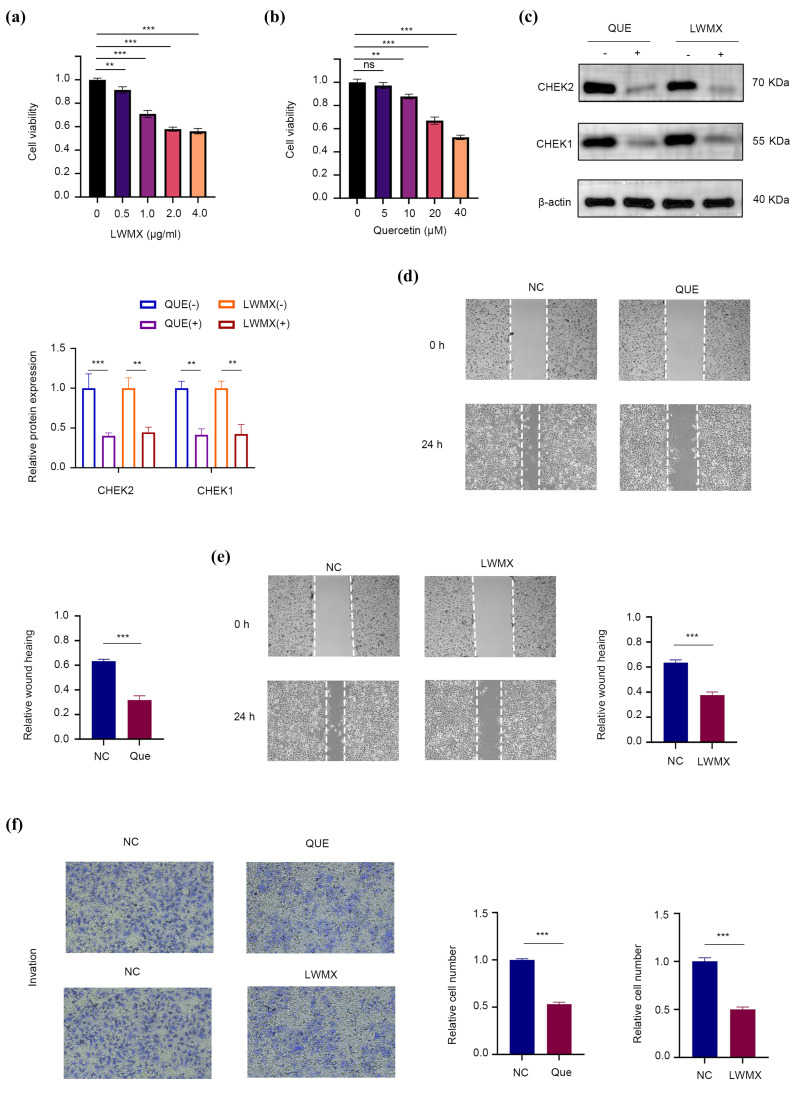
Inhibitory effects of LWMX and QUE on Huh7 cells. (**a**,**b**) Dose-dependent inhibition in cell proliferation assay. (**c**) Decreased CHK1/2 protein expression in Western blot analysis with β-actin as control. (**d**,**e**) Significantly reduced cell migration in scratch assay after treatment. Magnification: ×200. (**f**) Significant decrease in invasive cell counts by LWMX and QUE. Magnification: ×200. ** *p* < 0.01, *** *p* < 0.001, n.s. *p* > 0.05.

The results indicate that Liuwei Muxiang Pill (LWMX) and its active component quercetin (QUE) exhibit significant inhibitory effects on Huh7 hepatocellular carcinoma cells. In the cell proliferation assay (Figure 9a,b), both LWMX (0.5–4.0 μg/mL) and QUE (5–40 μM) demonstrated a dose-dependent inhibitory effect, with the survival rates of the highest concentration groups reduced to 56% and 52%, respectively (*p* < 0.01). Western blot analysis (Figure 9c) revealed that the QUE and LWMX treatment groups significantly decreased the expression of CHK1/2 proteins compared to the untreated group (*p* < 0.01), with β-actin used as a stable internal control. The scratch assay (Figure 9d,e) showed that the cell migration ability was significantly reduced after 24 h of treatment with QUE and LWMX compared to the NC group (*p* < 0.001), with a marked decrease in the filling of the scratch area. These findings suggest that LWMX may inhibit the proliferation and migration of hepatocellular carcinoma cells by downregulating CHK1/2 expression. Figure 9f reveals that, in comparison to the control group NC, both the quercetin-treated group (Que) and the Tibetan herbal medicine Liuwei Muxiang pill-treated group (LWMX) exhibited a significant reduction in the rate of cell invasion (*p* < 0.001). This suggests that both quercetin and the Liuwei Muxiang pill are effective in inhibiting the invasive capabilities of Hepatocellular Carcinoma Cells Huh7. In vitro experiments showed that either sorafenib or LWMX alone significantly inhibited Huh7 cell proliferation (Supplementary Material Figure S7), reducing cell viability to approximately 50%, with sorafenib exhibiting stronger inhibition. Combination treatment further reduced cell viability to 30%, significantly lower than the expected additive effect (~40%), indicating synergistic interaction (CI < 1).

## 3. Discussion

This study analyzes the mechanisms through which the Tibetan medicine Liuwei Muxiang pill (LWMX) and its active component quercetin (QUE) may exert anti-hepatocellular carcinoma (HCC) effects. Utilizing network pharmacology, machine learning, molecular docking, dynamic simulation, and experimental validation, this study identified key molecular targets and pathways associated with the anti-HCC activity of LWMX. The results underscore the critical involvement of CHK1 and additional core genes (CCNA2, CHK2, E2F1, and TOP2A) in the progression of HCC, indicating their potential as therapeutic targets. The research demonstrated that LWMX and its active ingredient quercetin markedly suppress HCC cell proliferation and migration, mainly by downregulating CHK1/2 expression. Molecular docking and dynamics simulations indicated significant binding affinities between quercetin and CHK1, implying a potential mechanism for the observed anti-HCC effects. Furthermore, five essential genes (CCNA2, CHK1, CHK2, E2F1, and TOP2A) were identified as closely linked to HCC progression and patient prognosis. The findings establish a molecular foundation for the therapeutic potential of LWMX in hepatocellular carcinoma (HCC) treatment and present new insights into the role of CHK1 in HCC pathogenesis.

The results are consistent with earlier research that has identified CHK1 as a factor in cancer progression, especially concerning DNA damage response and cell cycle regulation. CHK1 plays a crucial role in maintaining genomic stability, with its overexpression noted in several cancers, including HCC [12,13]. The findings support the conclusion that CHK1 is significantly upregulated in HCC tissues and that its inhibition by quercetin results in decreased HCC cell proliferation and migration. This indicates that CHK1 may serve as a viable therapeutic target for HCC, aligning with recent investigations into CHK1 inhibitors in cancer treatment [14].

Quercetin, a natural flavonoid, has undergone significant investigation regarding its anti-cancer properties. Molecular docking and dynamics simulations demonstrated that quercetin exhibits strong binding affinity to CHK1, thereby stabilizing the protein–ligand complex and potentially inhibiting its function. This aligns with prior research demonstrating quercetin’s capacity to induce cell cycle arrest and apoptosis in cancer cells [15,16]. The study offers new insights into the interaction between quercetin and CHK1, suggesting a potential mechanism for its anti-HCC effects. This contributes to the increasing evidence base for quercetin as a natural anti-cancer agent. To validate the anti-HCC potential of Liuwei Muxiang pill, the findings were compared with studies on quercetin-containing traditional Chinese medicine (TCM) compounds. Quercetin, as a core component of various TCM formulas, demonstrated significant antitumor effects. For example, the Xihuang pill was reported to inhibit HCC cell proliferation by regulating autophagy and macrophage polarization [6]; in silico and in vitro experiments confirmed quercetin’s critical role in the anti-HCC activity of Yiqi Jianpi Jiedu Formula [17]; multilevel analysis predicted the therapeutic potential of the Bupleuri Radix–Rhizoma compound against HCC cells, with quercetin identified as a key active component; the Shufeng Jiedu capsule (containing quercetin) was shown to induce apoptosis, inhibit migration and invasion, while enhancing doxorubicin efficacy [18]; and computational pharmacology studies revealed that quercetin and other components in Alchornea laxiflora possessed anti-HCC potential [19]. These studies collectively confirmed quercetin’s anti-HCC effects from multiple perspectives, aligning with the findings on the Liuwei Muxiang pill and supporting its therapeutic value for HCC treatment.

The identification of CCNA2, CHK1, CHK2, E2F1, and TOP2A as core genes in HCC is consistent with previous research that has linked these genes to cancer progression. For instance, E2F1 is a well-known transcription factor involved in cell cycle regulation, and its overexpression has been associated with poor prognosis in HCC [20]. Similarly, TOP2A, a key enzyme in DNA replication, has been implicated in HCC progression and resistance to chemotherapy [21]. This study confirms the significance of these genes in HCC and offers a detailed analysis of their interactions and potential as therapeutic targets.

The study offers important insights into the anti-HCC mechanisms of LWMX and quercetin; however, several limitations must be acknowledged. The findings predominantly rely on in vitro experiments and computational simulations, which may not adequately reflect the complexity of HCC in vivo. Subsequent research should incorporate in vivo models to confirm these findings. Secondly, while CHK1 has been recognized as a significant target, the precise molecular mechanisms through which quercetin inhibits CHK1 activity have yet to be completely clarified. Additional experimental investigations are required to examine this interaction more thoroughly.

Notwithstanding these limitations, the study possesses several strengths. A multi-disciplinary approach was utilized, integrating network pharmacology, machine learning, and experimental validation, to thoroughly examine the anti-HCC effects of LWMX. This integrative approach facilitated the identification of essential molecular targets and pathways, establishing a robust foundation for subsequent research. The application of molecular dynamics simulations to investigate the interaction between quercetin and CHK1 provides a new insight into the possible mechanisms underlying quercetin’s anti-cancer properties.

While our current study provides compelling in vitro and computational evidence supporting the anti-HCC potential of the Tibetan medicine Liuwei Muxiang pill and its active component quercetin, we acknowledge that these findings would benefit from further validation in physiologically relevant models. Future studies employing murine xenograft models would be valuable to assess the anti-tumor efficacy of these compounds in a more complex biological system that accounts for pharmacokinetic parameters, host–tumor interactions, and potential systemic effects [22,23]. Such in vivo investigations could provide critical translational insights regarding dosage optimization, biodistribution, and potential off-target effects, ultimately bridging the gap between our current findings and clinical application. These planned studies will build upon the mechanistic understanding established in the present work while addressing the important physiological context noted in previous HCC therapeutic development.

## 4. Materials and Methods

### 4.1. Screening of Common Targets for Drugs and Hepatocellular Carcinoma

The study referred to the document with the DOI: 10.1016/j.phymed.2023.154826, which identified the chemical components of the Tibetan medicine Liuwei Muxiang pill (produced by Ganlu Tibetan Medicine Co., Ltd., Lsaha, China) using ultra-high-performance liquid chromatography–quadrupole–Orbitrap mass spectrometry (UPLC-Q-Exactive Orbitrap MS). The equipment was sourced from Thermo Scientific, located in Waltham, MA, USA. The literature (doi: 10.3390/ph17040429) offers a comprehensive account of the prescription composition of the Tibetan medication Liuwei Muxiang Wan, the target genes modulated by these prescriptions, and the principal active constituents they encompass [3]. The conventional formulation of this medication has six Chinese herbal components: Aucklandiae Radix (Aucklandia), Phyllanthi Fructus (*Phyllanthus emblica*), Amomi Fructus Rotundus (round cardamom), Piperis Longi Fructus (long pepper), *Punica granatum* (pomegranate), and *Veronica eriogyne*. The research has pinpointed its principal active components, namely quercetin, (-)-epigallocatechin-3-gallate, luteolin, and palmitoleic acid. These components may exhibit potential bioactivity; nevertheless, their precise mechanisms of action in vivo are not well understood. A thorough investigation was performed to identify the target genes regulated by these components, utilizing Sankey diagrams to visually represent the links between the therapeutic components and active molecules. The illustrations utilize various colored blocks to signify distinct medication components, while the lines explicitly illustrate the relationships between these components and target genes. To thoroughly elucidate the molecular attributes of hepatocellular carcinoma (HCC), genetic data from six reputable databases (DrugBank, GeneCards, CTD, OMIM, PharmGKB, and TTD) were amalgamated [24,25,26,27,28,29]. Genes unique to HCC were identified using the terms “hepatocellular carcinoma” or “HCC,” with duplicate gene names eliminated. Gene set enrichment analysis (GSEA) was subsequently utilized to uncover biological pathways significantly linked to HCC, offering crucial background information for further investigation [30]. Differential gene expression analysis was subsequently conducted on the GSE135631 dataset to pinpoint critical genes in HCC [31]. Following quality control and data normalization utilizing DESeq2 and edgeR tools, differentially expressed genes (DEGs) with |log2 fold change| > 1 and *p*-value < 0.05 were identified. Volcano plots and heatmaps were employed to visually represent the expression patterns of these genes, highlighting genes that were highly elevated or downregulated in HCC.

A weighted gene co-expression network (WGCNA) was established to investigate gene connections further [32]. The connection patterns of the network were evaluated by evaluating power values from 1 to 30, and the relationships between nodes were measured using correlation coefficients. Scatter plots depicting correlation coefficients across power levels were created to identify the best soft threshold and to validate the scale-free topology of the network. Hierarchical clustering techniques were employed to categorize genes into modules, and DynamicTreeCut (version 1.63-1) was utilized to enhance the module assignments. The Pearson correlation coefficients between gene significance (GS) and module membership (MM) were computed, and their associations were illustrated using scatter plots. Core genes in HCC were identified by integrating HCC target genes from six databases, differentially expressed genes (DEGs), and disease-specific genes obtained from WGCNA. A Venn diagram analysis was used to discover overlapping genes among these datasets, potentially serving as major regulators of HCC. This comprehensive research not only elucidated putative molecular causes of HCC but also offered essential insights for eventual therapeutic target identification.

Enrichment studies were performed utilizing cross-target data, employing Disease Ontology (DO), Gene Ontology (GO) for biological pathways, and the Kyoto Encyclopedia of Genes and Genomes (KEGG) [33,34,35]. This analysis utilized a set of R packages, including BiocManager (version 3.21), ClusterProfiler (version 4.16.0), AnnotationHub (version 3.16.0), org.Hs.eg.db (version 3.16.0), pathview (version 1.48.0), dplyr (version 1.1.4), DOSE (version 4.2.0), and ggplot2 (version 3.5.2). A *p*-value cutoff of 0.05 was used for screening (*p* < 0.01 was considered indicative of significant enrichment, with lower *p*-values corresponding to higher enrichment levels). The results were visualized using Sankey bubble plots and bar charts. The STRING database https://string-db.org/ (accessed on 3 April 2025) was used to investigate the targets of Liuwei Muxiang pill (LWMX) in HCC treatment, with a confidence threshold set above 0.4 [36]. The top 15 key genes were identified using topological algorithms in Cytoscape 3.9.0 and CytoHubba (version 0.1). Genetic interaction networks for the five key genes and their neighboring genes were constructed using GeneMANIA database https://genemania.org/ (accessed on 3 April 2025) [37]. GO and KEGG enrichment analyses of the core genes were performed using ClusterProfiler (version 4.16.0).

### 4.2. Multi-Gene Analysis of Core Targets: A Systematic Exploration from Molecular Mechanisms to Clinical Implications

#### 4.2.1. Molecular Localization and Construction of Protein–Protein Interaction Networks

Initially, subcellular localization analysis was conducted on five core genes using the GeneCards database to delineate their distribution characteristics within cells. Subsequently, interaction information among the proteins encoded by these genes was retrieved from the STRING database, and the network layout was optimized using Cytoscape to more clearly illustrate the relationships between nodes. Additionally, based on the TargetScan https://www.targetscan.org (accessed on 4 April 2025), miRDB https://mirdb.org/ (accessed on 4 April 2025), and miRanda http://www.microrna.org/ (accessed on 4 April 2025) databases, a ceRNA regulatory network for these genes was predicted and constructed, revealing their potential roles in post-transcriptional regulation.

#### 4.2.2. Clinical Correlation Analysis: From Gene Expression to Patient Prognosis

To explore the clinical significance of these genes in hepatocellular carcinoma (HCC), the study utilized the TCGA-LIHC dataset to analyze the relationships between CCNA2, CHK1, CHK2, E2F1, and TOP2A and patient age, gender, tumor stage (TNM stage), and tumor size (T) [38]. Further, using the UALCAN database, the expression differences of these genes between HCC tissues and normal tissues were compared, revealing significant upregulation in HCC tissues, suggesting their potential as biomarkers [39]. Using the GSE101685 dataset, differential expression analysis was performed to systematically evaluate the expression levels of five core genes (CCNA2, CHK1, CHK2, E2F1, and TOP2A) in eight normal liver tissue samples and 24 hepatocellular carcinoma cell line samples. Moreover, survival analysis methods were employed to assess the relationship between the expression levels of these genes and the prognosis of HCC patients.

#### 4.2.3. Impact of Environmental Chemicals: From Gene-Environment Interactions

To further uncover external environmental factors contributing to HCC, the study utilized the Comparative Toxicogenomics Database (CTD) to analyze the associations between the five core genes and environmental chemicals related to HCC [26]. Through keyword searches and filtering, chemical–gene interaction data directly related to these genes were screened, and a “protein-HCC-environmental chemical” network was constructed using Cytoscape. Visualization through Sankey diagrams and network graphs clearly demonstrated the complex interactions between these genes and environmental chemicals, providing new insights into the environmental risk factors for HCC.

### 4.3. Molecular Docking

To investigate the interaction mechanisms between Tibetan medicine active ingredients and core target proteins, molecular docking technology was employed. Molecular docking is a key computer-aided drug design method that predicts binding modes (conformations) and affinity (binding energy) by simulating the binding process of small molecule ligands to biological macromolecules (such as proteins), thereby assessing potential activity. Four representative active ingredients from Tibetan medicine—epigallocatechin-3-gallate, luteolin, palmitoleic acid, and quercetin—were selected for docking studies against five core target proteins (CCNA2, CHK1, CHK2, E2F1, TOP2A).

The specific workflow was as follows: First, the crystal structures of the five target proteins (PDB IDs: 6ATH, 4HYI, 2YCF, 6G0P, 6ZY5) were downloaded from the RCSB Protein Data Bank (PDB). To enhance docking accuracy, the protein structures were pre-processed using PyMOL software (version 3.0.3), including the removal of crystallographic water molecules, original ligands, and any potential non-natural modifications. Subsequently, the processed protein structures were imported into AutoDock Tools 1.5.7 software for hydrogenation (adding polar and nonpolar hydrogens) and charge calculation (using Gasteiger charges).

Reliable results depended critically on docking parameter settings. Based on the size of the target proteins’ active sites and literature reports, the Grid Box size (e.g., center coordinates, extension range in X/Y/Z directions) was appropriately defined, and suitable genetic algorithm parameters (such as population size, number of iterations) were selected. Docking calculations were performed by running AutoDock Vina via the command line.

Finally, PyMOL was utilized to visualize and analyze the docking results. Specific interactions between ligands and target proteins—such as hydrogen bonds, hydrophobic interactions, and π-π stacking—were observed. The predicted binding energy (ΔG) for each ligand–target protein pair was recorded. This value, typically used to assess binding affinity, indicates stronger affinity with increasingly negative values.

### 4.4. 113 Diagnostic and 101 Prognostic Machine Learning Ensemble Algorithms

The gene expression profile data of HCC were systematically analyzed using machine learning methods to construct diagnostic and prognostic models. The GSE144269 dataset was used as the training set, and the TCGA-LIHC dataset was used as the validation set to construct 113 different diagnostic machine learning combination algorithms. These algorithms combined multiple classifiers, such as logistic regression (LASSO), support vector machine (SVM), random forest (RF), etc., as well as different feature selection methods and model fusion strategies. The TCGA-LIHC dataset was split into a training set and a validation set to construct 101 prognostic machine learning combination algorithms. The intersection of the results obtained from the two optimal algorithms was taken to hopefully identify genes with both diagnostic and prognostic value.

### 4.5. Investigation of the Role of CHK1 in the Pan-Cancer Immune Microenvironment

To comprehensively explore the potential role of CHK1 in the pan-cancer immune microenvironment, a multi-layered integrative analysis was conducted based on the TCGA pan-cancer dataset. This analysis encompassed immune infiltration, immune inflammation, immune checkpoint correlations, as well as CHK1-associated immune evasion and therapeutic evaluation. Initially, a systematic assessment of the relationship between CHK1 expression and immune cell infiltration within the tumor microenvironment was performed using various immunological algorithms, including CIBERSORT, MCP-counter, xCell, TIMER, and ESTIMATE. These algorithms provided a multi-dimensional perspective on the composition of immune cells in the tumor microenvironment through different computational models, thereby offering a more comprehensive understanding of the potential associations between CHK1 expression and immune infiltration.

Further analysis focused on key signaling pathways related to immune inflammation, such as B-cell receptor signaling, chemokine signaling, and the complement/coagulation cascade. The association between CHK1 expression and the activity scores of these immune-inflammatory pathways was quantified by calculating Pearson correlation coefficients and assessing their significance (*p*-values). This analysis not only revealed the potential role of CHK1 in the regulation of immune inflammation but also provided new insights into its function within the tumor microenvironment.

Additionally, Pearson correlation analysis was employed to correlate CHK1 expression with the expression of immune checkpoint genes in the TCGA pan-cancer dataset, aiming to uncover the potential mechanisms of CHK1 in the development of hepatocellular carcinoma (HCC). By integrating the TCGA-LIHC (Liver Hepatocellular Carcinoma) expression profile data, the TIDE scoring system was further applied to evaluate the relationship between CHK1 expression and immune evasion as well as the efficacy of immunotherapy. Through the integration of single-gene CHK1 expression data from both normal and tumor groups, combined with TIDE scores, a more comprehensive assessment of the potential role of CHK1 in tumor immune evasion and response to immunotherapy was achieved.

To investigate the role of CHK1 in tumor immune microenvironment and its relationship with CD4 T cells and immune checkpoints like PD-L1, literature was first searched through PubMed. One study revealed that decreased CHK1 levels upregulated PD-L1 expression in hepatocellular carcinoma by increasing STAT3 phosphorylation [40]. Subsequently, GSEA analysis was performed using the public HCC dataset GSE76427 to validate the association between CHK1 and immune-related pathways.

### 4.6. Analysis of CHK1 and Drug Sensitivity in Hepatocellular Carcinoma

In this study, the role of CHK1 in the drug sensitivity of HCC was investigated, and the CB·DOCK2 online platform was utilized for molecular docking experiments to uncover the interactions between the CHK1 protein and various drug molecules [41]. At the outset, data on the correlation between the CHK1 gene and GDSC drug sensitivity and expression were retrieved from the GSCA database [42]. Through comprehensive analysis, drugs with significant positive and negative correlations with CHK1 expression were identified. To further explore the potential binding patterns of these drugs with the CHK1 protein, the molecular docking service provided by the CB·DOCK2 website was employed. CB·DOCK2 is an efficient online molecular docking tool that predicts the likelihood of interactions by calculating the binding energy between drug molecules and target proteins. In the experiment, the three-dimensional structure of the CHK1 protein was first uploaded, and the chemical structure information of the selected drug molecules was inputted. CB·DOCK2 then automatically performed the docking process, which included a conformational search of the drug molecules, identification of binding sites, and calculation of binding energy.

### 4.7. Molecular Dynamics Simulation of CHK1 and Quercetin Complex

Molecular dynamics simulations were performed to analyze CHK1 protein, quercetin, and their complex [43]. Structural data were collected and RMSD values calculated at various time points. The CHK1 structure from PDB was preprocessed using GROMACS (hydrogen addition, topology definition, solvent environment setup). The AMBER force field was applied for simulation, while the GAFF force field with quantum chemical calculations parameterized quercetin. After energy minimization and NVT/NPT ensemble equilibration, 100 ns production simulations generated trajectory data for RMSD/RMSF analysis. Hydrogen bond dynamics between CHK1 and quercetin were statistically analyzed, including temporal distribution patterns and stability assessment. SASA variations and radius of gyration (Rg) profiles were monitored throughout simulations. Conformational stability was evaluated through Gibbs free energy landscape construction using metadynamics. Free energy contribution heatmaps were generated via MM-PBSA calculations to identify key interacting residues. All analyses incorporated statistical evaluation of fluctuation ranges and average values to assess structural dynamics.

### 4.8. IHC and qRT-PCR in HCC Samples

This study utilized clinical samples of HCC as research subjects, employing immunohistochemistry (IHC) and qRT-PCR techniques for cross-validation [44,45]. Human tissues were sourced from HCC and adjacent normal liver tissue samples provided by the First Affiliated Hospital of Wenzhou Medical University. The use of all clinical specimens was reviewed and approved by the hospital’s Clinical Research Ethics Committee (Ethics Number: KY2023-198). Sample collection and processing strictly adhered to aseptic procedures. The resected tumors and corresponding normal liver tissues were processed through fixation, embedding, and other procedures to prepare paraffin sections. For immunohistochemical detection, CHK1 antibody was used as the core reagent. The standardized process includes dewaxing and rehydration, antigen retrieval, blocking, antibody incubation, DAB chromogenic reaction, and hematoxylin restaining. Positive results were determined by the presence of brownish-yellow granules in the cytoplasm/nucleus.

For qRT-PCR detection, total RNA was extracted from the liver tissues of hepatocellular carcinoma group and the normal control group using the Trizol method. After the concentration and purity were verified as acceptable by NanoDrop 2000 (Thermo Scientific, Waltham, MA, USA), cDNA was synthesized following the instructions of the reverse transcription kit. Specific primers for CHK1 were designed, and the reaction system was set up on the ABI StepOne Plus system (Applied Biosystems, Boston, MA, USA), which included SYBR Green Mix, cDNA, and primers. The program began with a 5 min pre-denaturation at 95 °C, followed by 40 cycles of 95 °C for 15 s, 60 °C for 30 s, and 72 °C for 30 s amplification. Finally, a melting curve was performed to verify specificity. Relative gene expression was calculated using the 2-ΔΔCt method, and all data were statistically analyzed by *t*-test using GraphPad Prism software (version 10.1), with *p* < 0.05 as the threshold for significance. To verify the effect of Tibetan medicine Liuwei Muxiang pills on the expression level of the CHK1 gene, quantitative real-time polymerase chain reaction (qPCR) was conducted. The experiment was divided into two groups: the control group (untreated with the drug) and the drug-treated group (treated with Tibetan medicine Liuwei Muxiang pills).

### 4.9. Assessing Huh7 Cell Response to LWMX and Quercetin via CCK-8 Assay and Functional Tests

Investigating the Mechanism of Action of Liuwei Muxiang pill (LWMX) and Its Active Component Quercetin (QUE) on Hepatocellular Carcinoma Cells Huh7 Using a Multidimensional In Vitro Experimental System [46]. Initially, the CCK-8 assay was employed to assess the impact of the drugs on cell proliferation, with Huh7 cells exposed to LWMX at concentrations ranging from 0–4.0 μg/mL or QUE in the environment at doses from 0–40 μM for a continuous period of 48 h, followed by a quantitative analysis of changes in cell viability. To delve into the target of action, Western Blotting techniques were used to compare the expression differences of key DNA damage response proteins CHK1/CHK2 among the control groups, the LWMX group (2 μg/mL LWMX treatment), and the QUE group (30 μM QUE treatment). In the assessment of cell migration capacity, a scratch assay was conducted for the four groups. Images were captured using microscopy at 0 h and 24 h after wounding. Hepatocellular carcinoma cells, Huh7, were cultured under standard conditions and divided into three groups: a control group (NC), a quercetin treatment group (Que), and a treatment group with the Tibetan herbal formula Liuwei Muxiang pill (LWMX). After 24 h of treatment with quercetin and Liuwei Muxiang pill in the Que and LWMX groups, respectively, the cell invasion capabilities were assessed using Transwell chamber assays. For the invasion assay, cells were seeded onto the upper chamber of the Transwell with a Matrigel coating. Following treatment, cells were stained with crystal violet and counted under a microscope [47]. Each experiment was repeated at least three times, and the data are presented as mean ± standard deviation. Statistical analysis was performed using Student’s *t*-test.

To investigate the potential synergy between the Tibetan medicine Liuwei Muxiang pill (LWMX), its main component quercetin, and the standard HCC drug sorafenib, in vitro experiments were conducted using HCC cells. Cells were divided into four groups: control (medium only), sorafenib alone, LWMX alone, and combination treatment. Drug concentrations were selected based on prior studies and clinically effective ranges, with combination doses set at partial inhibition levels.

Cell viability was measured by MTT assay. After incubation with MTT reagent, formazan crystals were dissolved in DMSO, and absorbance at 570 nm was recorded to calculate survival rates (triplicate experiments). The interaction type (synergistic, additive, or antagonistic) was quantified using the Combination Index (CI), computed via the Chou–Talalay method (CompuSyn software version 10.1). A CI < 1 indicated synergy, CI = 1 additive effects, and CI > 1 antagonism.

## 5. Conclusions

This study reveals that the Liuwei Muxiang pill (LWMX) exerts anti-HCC effects by targeting cell cycle regulation, with CHK1 identified as a key mediator. Multi-omics and experimental validation demonstrated that LWMX’s active component quercetin stably binds CHK1, inhibiting HCC proliferation and migration [48]. These findings position CHK1 as a potential therapeutic target and support LWMX’s clinical potential for HCC treatment.

## Data Availability

Public datasets were sourced from the Gene Expression Omnibus (GEO), The Cancer Genome Atlas (TCGA-LIHC), Comparative Toxicogenomics Database (CTD), and other integrated repositories.

## Supplementary Materials

The following supporting information can be downloaded at: https://www.mdpi.com/article/10.3390/ph18060900/s1. Figure S1: Mass spectrometric detection of four core active ingredients; Figure S2: Names and structures of the core active ingredients; Figure S3: ROC validation of the external dataset; Figure S4: Upregulated genes in HCC (GSE101685 dataset). Figure S5: Binding Energy Heatmap; Figure S6: The Tibetan medicine Liuwei Muxiang pills significantly downregulate CHK1 mRNA in HCC; Figure S7: The inhibitory effect of sorafenib combined with LWMX on the proliferation of Huh7 cells; Table S1: All abbreviations and corresponding full names; Table S2: Compound and protein information for molecular docking, as well as binding energy; Table S3: Results of GSEA enrichment analysis; ZIP: Molecular dynamics simulations results.
