# Supplementary figures and images for: Multi-Omics and Experimental Validation Reveal Anti-HCC Mechanisms of Tibetan Liuwei Muxiang Pill and Quercetin
Source: Pharmaceuticals (Basel). 2025 Jun 16;18(6):900. doi: 10.3390/ph18060900 (PMC12196418; doi:10.3390/ph18060900)

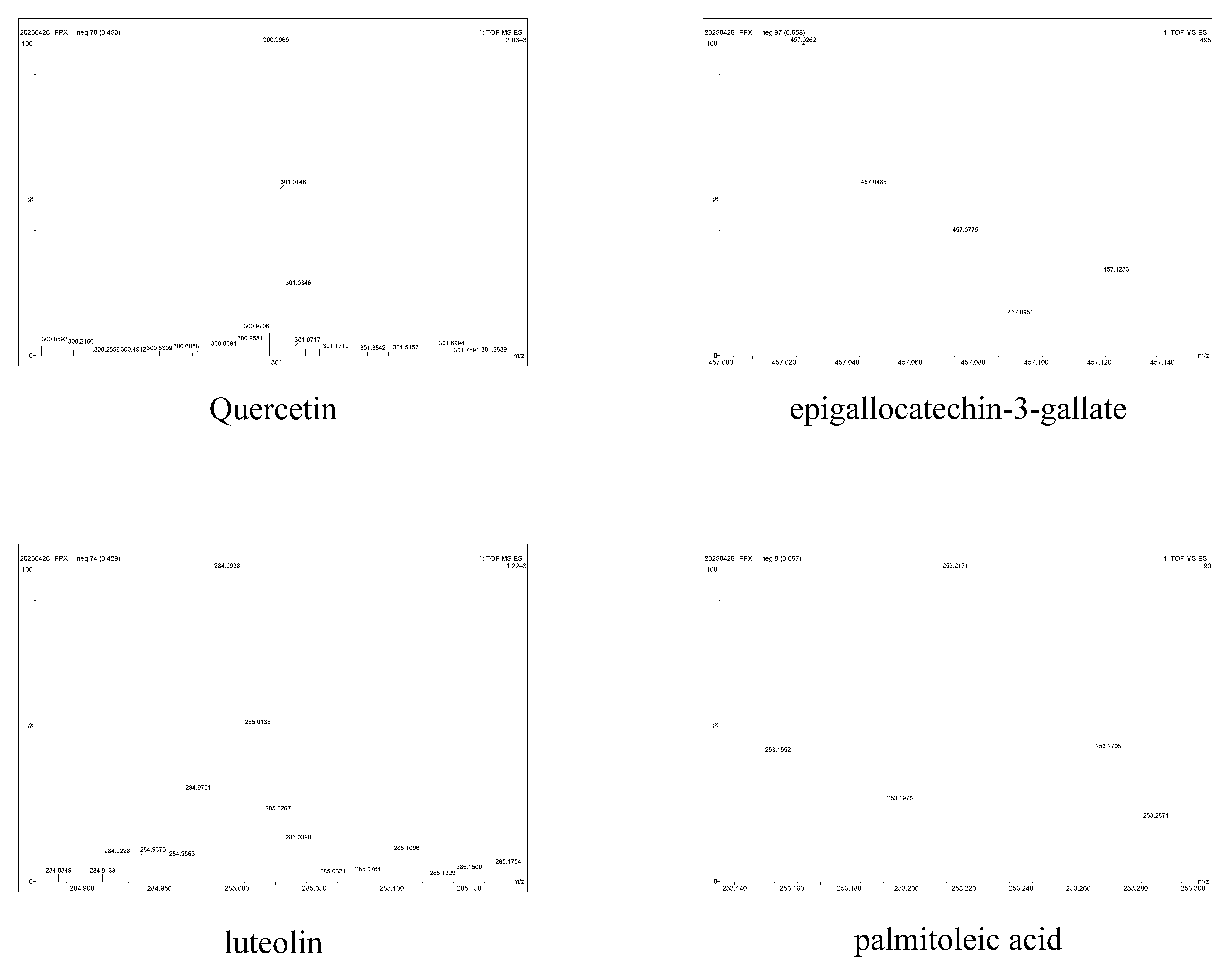

Supplement: Supplementary file 1 [file pharmaceuticals-18-00900-s001.zip › Figure S1.tif]

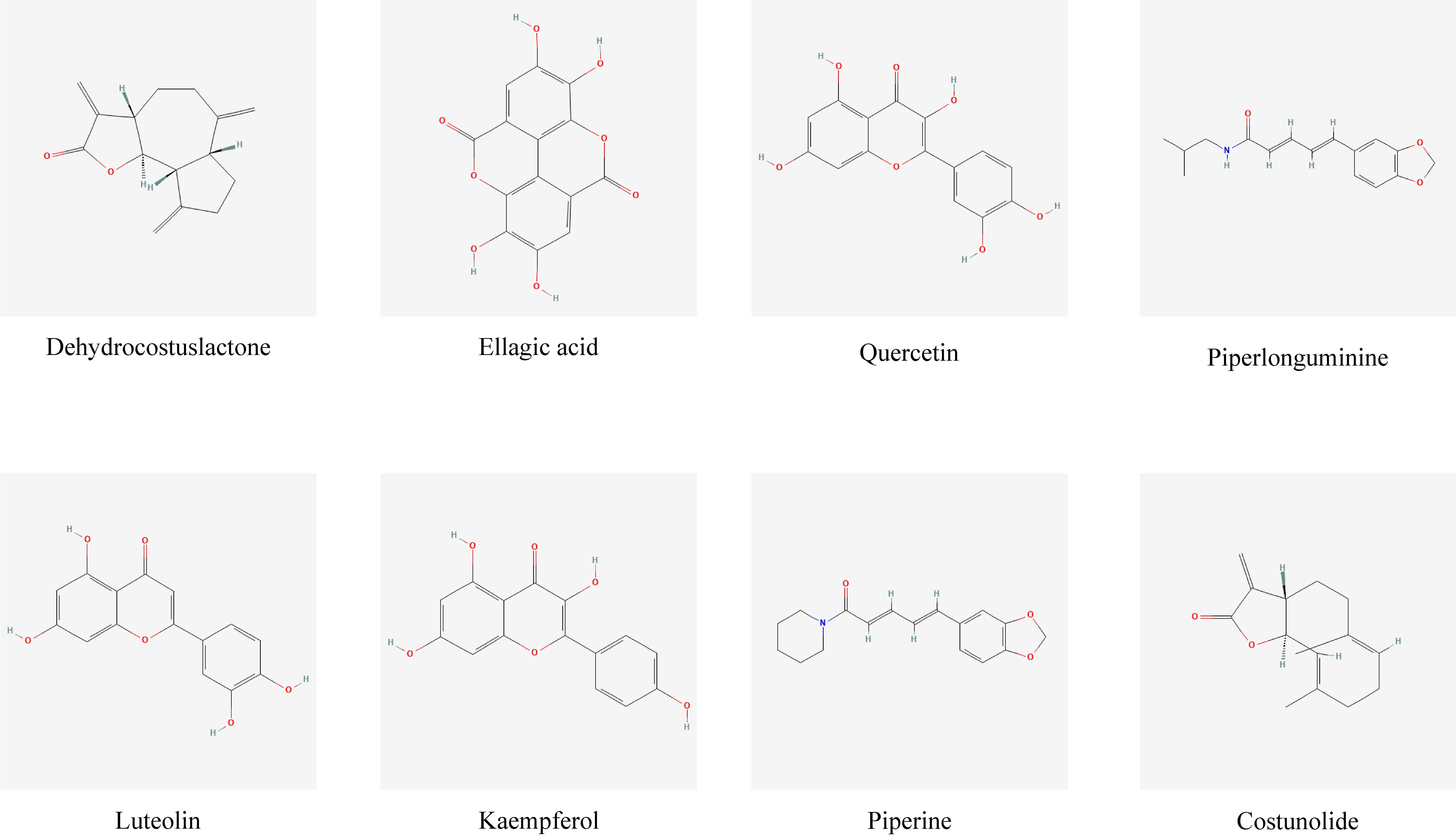

Supplement: Supplementary file 1 [file pharmaceuticals-18-00900-s001.zip › Figure S2.tif]

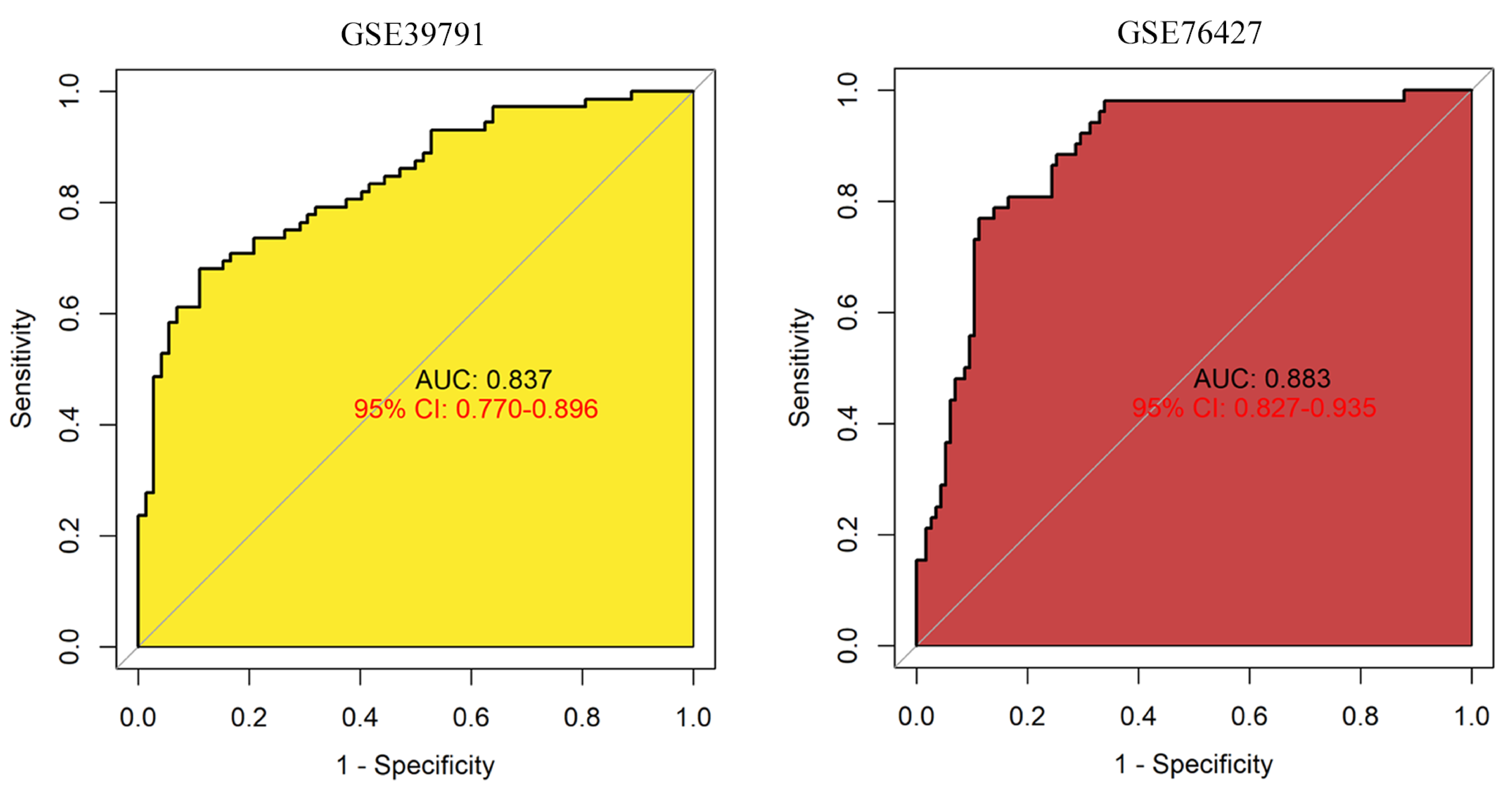

Supplement: Supplementary file 1 [file pharmaceuticals-18-00900-s001.zip › Figure S3.tif]

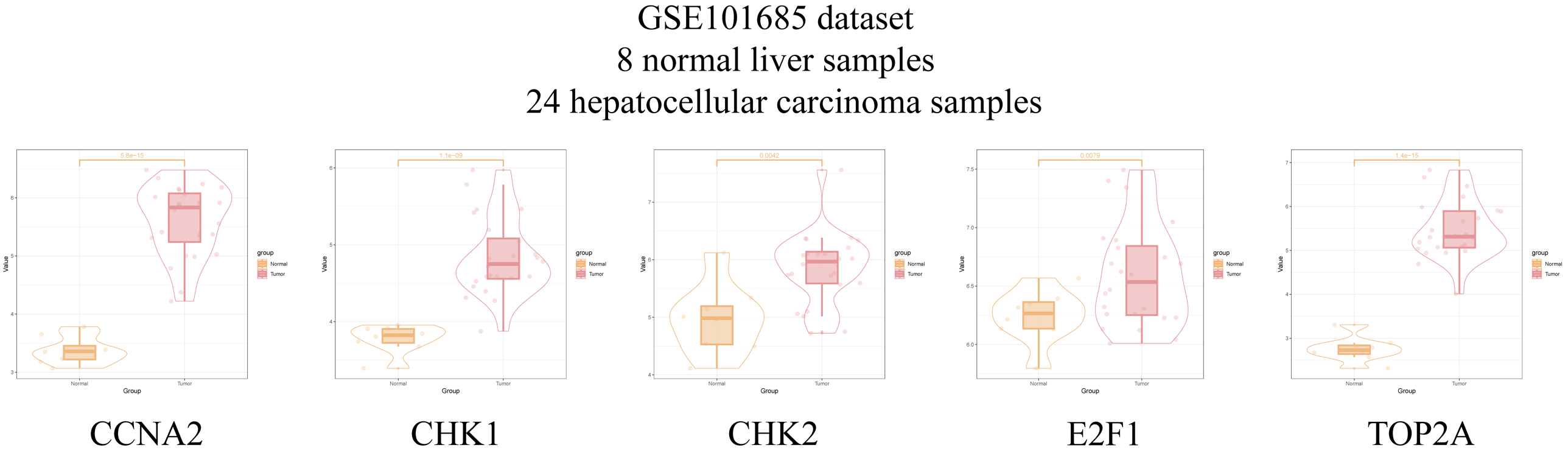

Supplement: Supplementary file 1 [file pharmaceuticals-18-00900-s001.zip › Figure S4.tif]

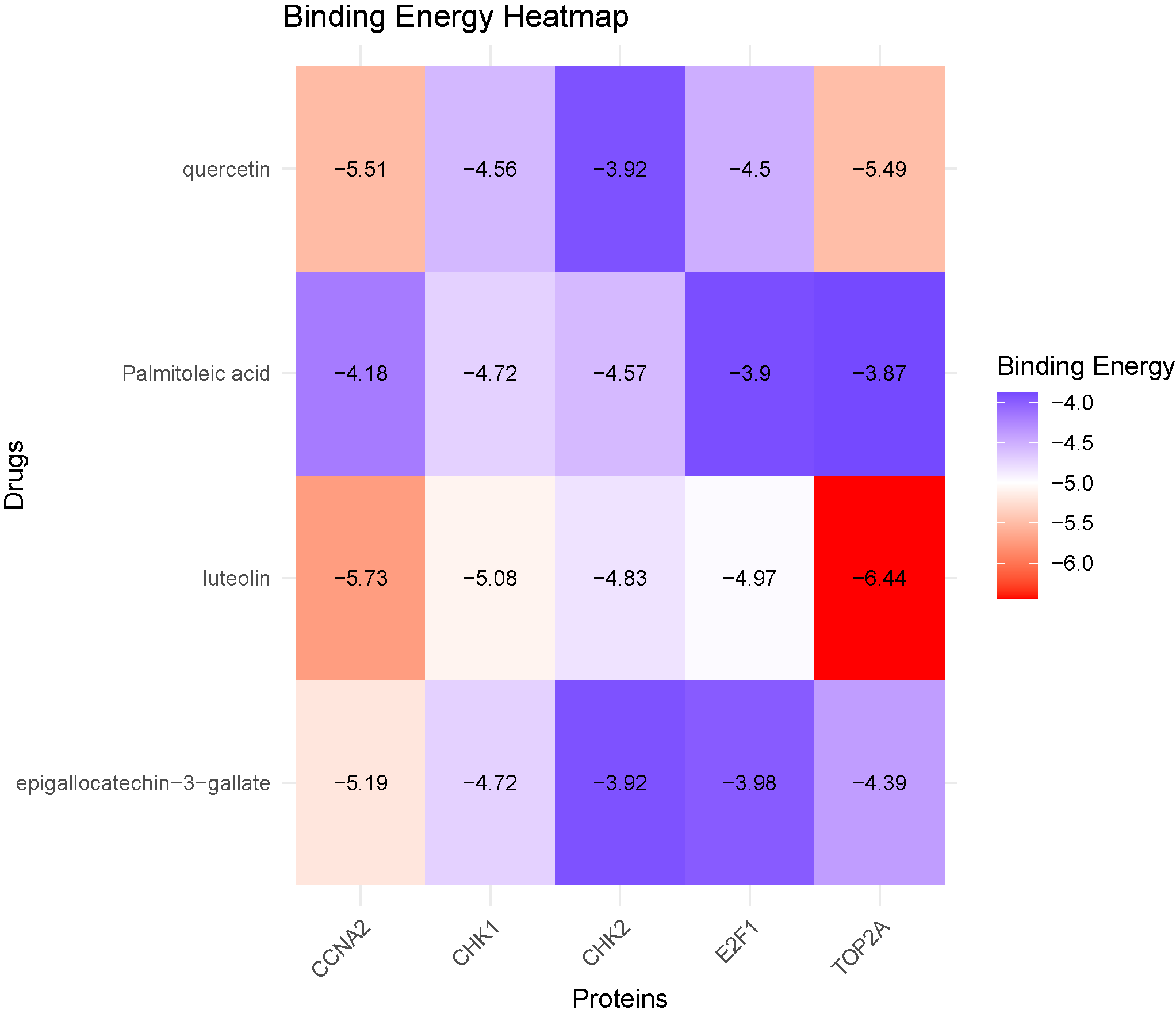

Supplement: Supplementary file 1 [file pharmaceuticals-18-00900-s001.zip › Figure S5.tif]

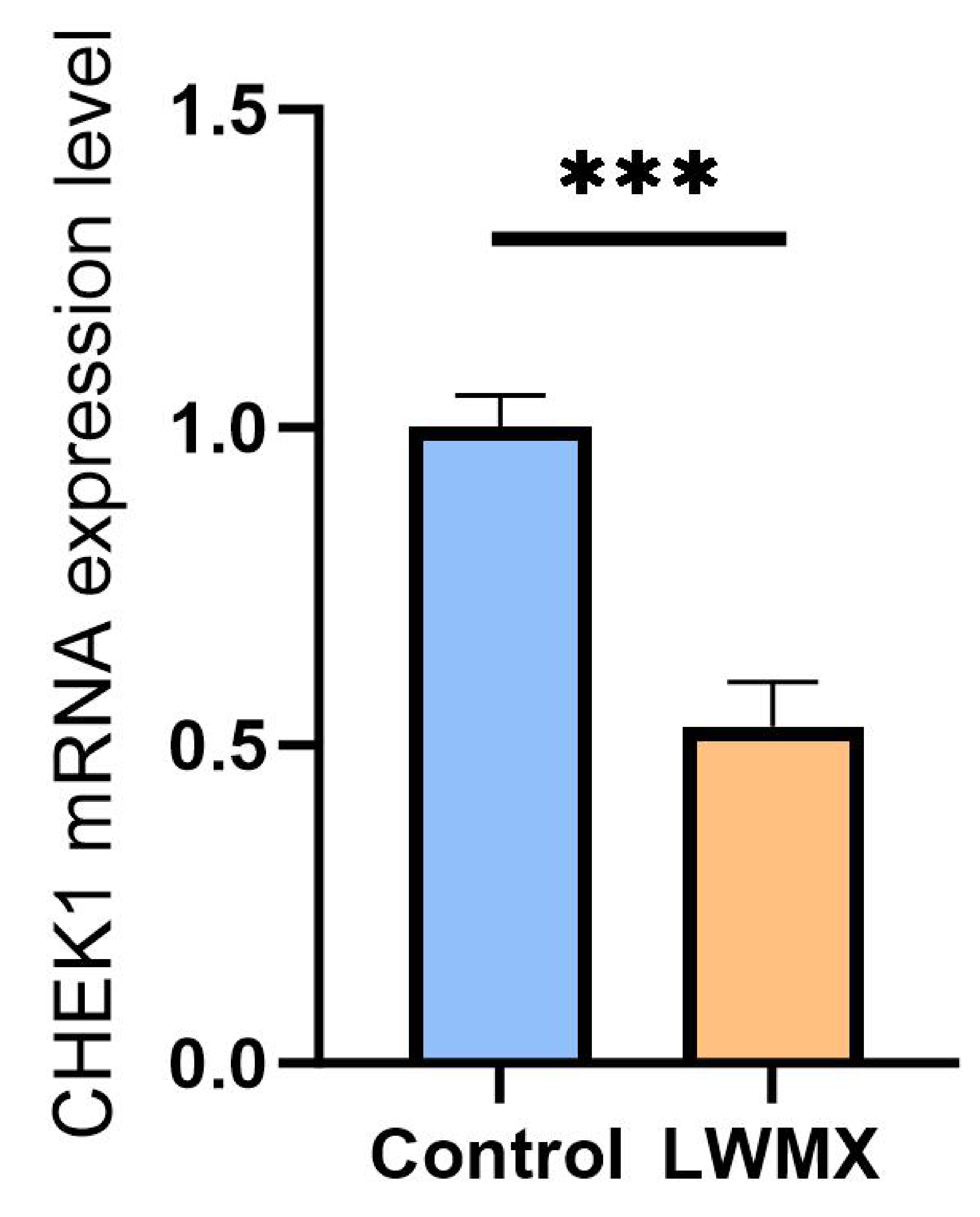

Supplement: Supplementary file 1 [file pharmaceuticals-18-00900-s001.zip › Figure S6.tif]

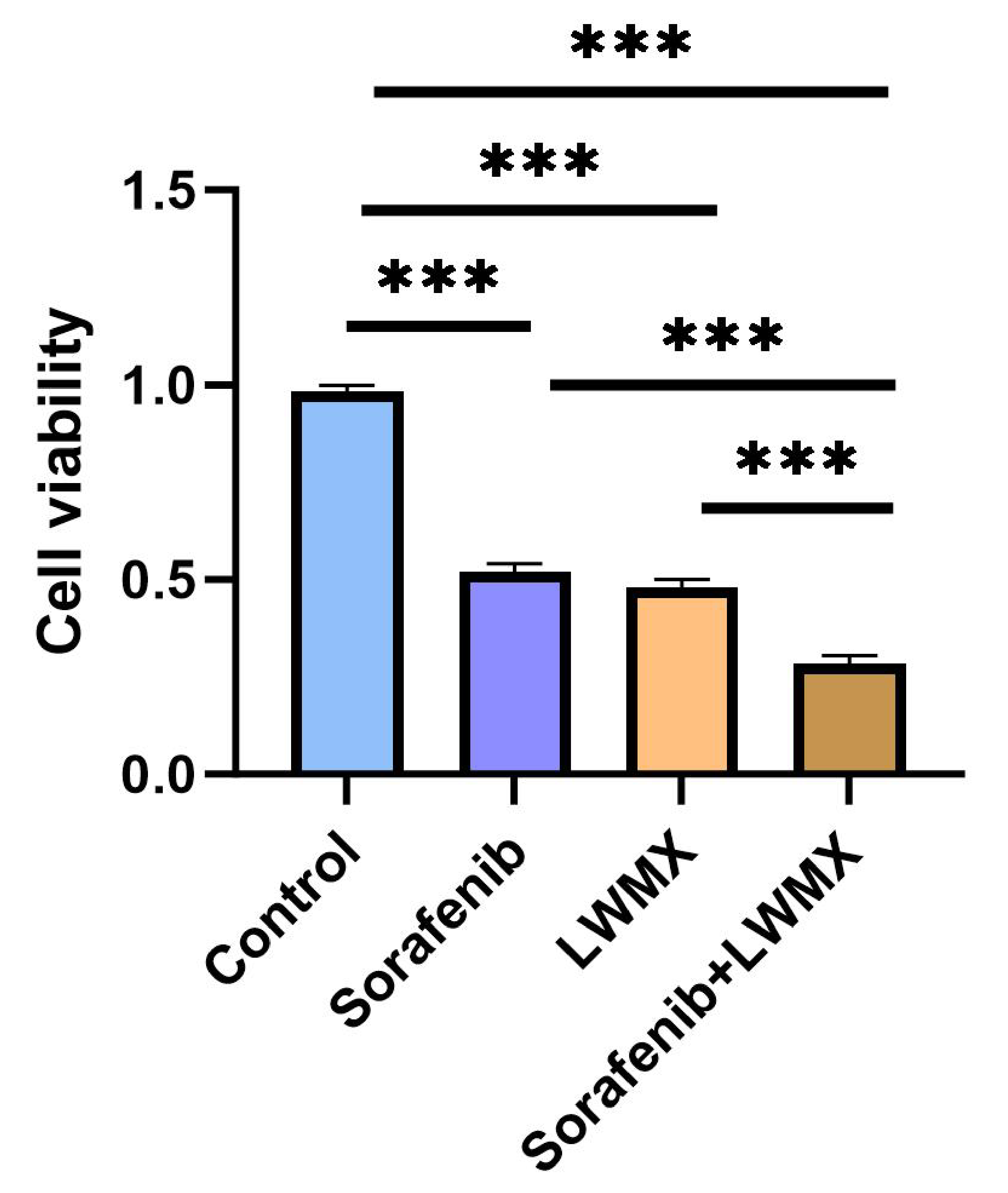

Supplement: Supplementary file 1 [file pharmaceuticals-18-00900-s001.zip › Figure S7.tif]
